# Setting Research Priorities to Reduce Global Mortality from Childhood Pneumonia by 2015

**DOI:** 10.1371/journal.pmed.1001099

**Published:** 2011-09-27

**Authors:** Igor Rudan, Shams El Arifeen, Zulfiqar A. Bhutta, Robert E. Black, Abdullah Brooks, Kit Yee Chan, Mickey Chopra, Trevor Duke, David Marsh, Antonio Pio, Eric A.F. Simoes, Giorgio Tamburlini, Evropi Theodoratou, Martin W. Weber, Cynthia G. Whitney, Harry Campbell, Shamim A. Qazi, and the WHO/CHNRI Expert Group on Childhood Pneumonia

**Affiliations:** 1 Centre for Population Health Sciences, The University of Edinburgh Medical School, Edinburgh, Scotland, United Kingdom; 2 Croatian Centre for Global Health, Faculty of Medicine, University of Split, Split, Croatia; 3 ICDDR, B Centre for Health and Population Research, Dhaka, Bangladesh; 4 Department of Paediatrics and Child Health, The Aga Khan University, Karachi, Pakistan; 5 Department of International Health, Johns Hopkins Bloomberg School of Public Health, Baltimore, Maryland, United States of America; 6 Nossal Institute for Global Health, Melbourne University, Melbourne, Australia; 7 Department of Health, UNICEF, New York, New York, United States of America; 8 Centre for International Child Health, University of Melbourne, Parkville, Australia; 9 Save the Children, Amherst, Massachusetts, United States of America; 10 Senior Advisor in Public Health and Respiratory Diseases, Mar de Plata, Argentina; 11 Department of Pediatrics Infectious Diseases, The Children's Hospital, Denver, Colorado, United States of America; 12 Institute for Child Health IRCCS Burlo Garofolo, Trieste, Italy; 13 WHO Country Office, Jakarta, Indonesia; 14 Centers for Disease Control and Prevention, Atlanta, Georgia, United States of America; 15 Department of Child and Adolescent Health and Development, World Health Organization, Geneva, Switzerland

## Abstract

Igor Rudan and colleagues report the results of their consensus building exercise that identified health research priorities to help reduce child mortality from pneumonia.

**Summary Points**

- This paper aims to identify health research priorities that could assist the rate of progress in childhood pneumonia mortality reduction globally, as set out in the United Nation's Millennium Development Goal 4.
- The authors applied the Child Health and Nutrition Research Initiative methodology for setting priorities in health research investments. The process was coordinated by the World Health Organization.
- Forty-five leading childhood pneumonia researchers suggested more than 500 research ideas, which were merged into 158 research questions that spanned the broad spectrum of epidemiological research, health policy and systems research, improvement of existing interventions, and development of new interventions.
- Within the short time frame in which gains were expected globally, the research priorities were dominated by health systems and policy research topics (e.g., studying barriers to health care seeking and access, as well as barriers to increased coverage with available vaccines; and evaluating the potential to safely scale up antibiotic treatment through community health workers).
- These were followed by epidemiological questions to identify the main gaps in knowledge (e.g., predictors of severe pneumonia that requires hospitalisation); priorities for improvement of the existing interventions (e.g., training of community health workers to recognise danger signs, refer, and treat sick children); and identifying cost reduction mechanisms for the available conjugate vaccines.
- Among the new interventions, the greatest support was shown for the development of low-cost conjugate vaccines and cross-protective common protein vaccines against the pneumococcus.

## Introduction

Pneumonia is the leading single cause of mortality in children aged less than 5 years with approximately 1.6 million children dying each year [1]. This accounts for almost one in five under-5 deaths worldwide. Furthermore, approximately 155 million new episodes of clinical pneumonia occur in children under 5 years of age annually [2]. It is estimated that 7%–13% of episodes are severe enough to be life-threatening and require hospitalisation [3]. Studies have identified *Streptococcus pneumoniae*, *Haemophilus influenzae*, and respiratory syncytial virus (RSV) as the main pathogens associated with severe childhood pneumonia [4]–[6]. Future studies, with new molecular techniques to detect infections due to a wider range of pathogens, will improve our understanding of the cause of pneumonia [7]. The leading risk factors contributing to pneumonia incidence are lack of exclusive breastfeeding, undernutrition, exposure to indoor air pollution, low birth weight, crowding, and absence of immunisation [3].

## Initiatives to Control Childhood Pneumonia

The United Nation's (UN) Millennium Development Goal 4 (MDG4) states that childhood mortality should be reduced by two-thirds between 1990 and 2015, but recent estimates show that the progress in mortality reduction has been disappointing in some countries [8],[9]. Key reasons are lack of knowledge on how to implement existing cost-effective interventions and to achieve greater coverage of these interventions in low-resource settings [10], and the need to develop new effective interventions to amplify case management and immunisation strategies. In an attempt to accelerate progress in tackling childhood pneumonia, two major initiatives have been taken. A Global Action Plan for the Prevention and Control of Pneumonia (GAPP) was launched late in 2009 by the World Health Organization (WHO) and UNICEF in collaboration with other global partners, with a multitude of aims and several ongoing activities (see Box 1) [11]. The second major initiative was the successful passage of a resolution on the prevention and control of childhood pneumonia at the 2010 World Health Assembly. The resolution calls on the WHO to strengthen human resources in tackling this problem and to create an international forum to coordinate action. It calls on WHO Member States to create evidence-based and multi-sectoral action plans and to monitor progress [12].

**Box 1. Global Action Plan for the Prevention and Control of Pneumonia (GAPP)**

GAPP aims to increase awareness of pneumonia as a major cause of child death, calls for the scaling up of the use of the interventions of proven benefit, and provides guidance on how this can be done. The GAPP calls to action a broad coalition of global and government policy-makers, donor agencies, and civil society. GAPP recommends that every child is protected against pneumonia through a healthy environment, and has access to preventive and treatment measures. The key GAPP strategies for treating, preventing, and protecting from pneumonia are case management at all levels, vaccination, prevention and management of HIV infection, improvement of nutrition and breastfeeding, reduction of low birth weight, and control of indoor air pollution. Furthermore, pneumonia is recognised as a common and serious consequence of pandemic influenza, and preparedness for pandemic influenza should include prevention and control of pneumonia [11].

## Mismatch of Pneumonia Mortality Burden and Research Investment

The positive initiatives need research and investment, but neither has been commensurate with the importance of pneumonia as the leading child killer [13]. It has been shown that the amount of available research funds per disability-adjusted life year (DALY) of pneumonia is orders of magnitude lower compared to many other diseases today [14],[15]. To assist policy-makers and donors alike in understanding the potential of different research avenues to contribute to reducing the burden of disease and disability, the Child Health and Nutrition Research Initiative (CHNRI) recently developed a methodology that allows systematic listing and transparent scoring of many competing research options, thus exposing their strengths and weaknesses [16]–[18]. The Department of Child and Adolescent Health and Development (CAH) of WHO has used this methodology to identify health research priorities to tackle all the major causes of child deaths, and some of the exercises have already been published [19]–[21]. In this paper, we present the results of the CHNRI research priority-setting process for childhood pneumonia.

## Methods

**Figure 1:**
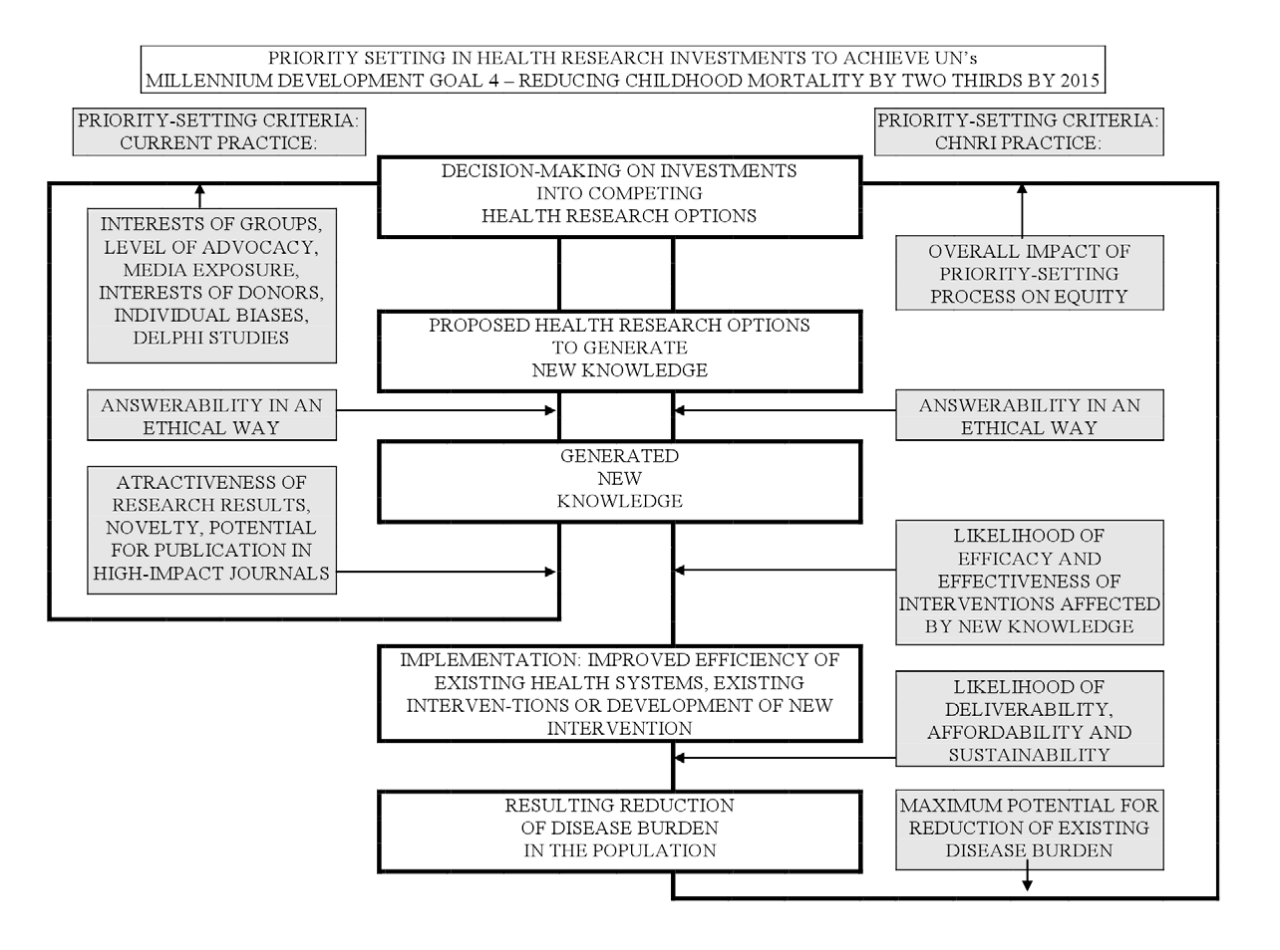
CHNRI's conceptual framework showing key steps required to get from investments in health research options to decrease in burden of death, disease, or disability. The framework identifies criteria to discriminate between likelihoods of success of competing research options: (i) answerability; (ii) effectiveness; (iii) deliverability; (iv) maximum potential for disease burden reduction; and (v) predicted impact on equity in the population (right side). These criteria are not necessarily what drives investment decisions in health research today (left side) [13],[16]–[18].

The CHNRI methodology for setting priorities in health research investments was proposed to inform those who develop research policy and/or invest in health research [16]–[18]. This aims to assist policy makers to understand the full spectrum of research investment options and the potential risks and benefits that can result from investments in different research. As shown in the published CONSORT diagram [13], the CHNRI methodology has four stages: (i) input from investors/policy-makers (who define the context and the criteria for priority setting); (ii) input from a larger group of technical experts (who propose, list systematically, and then independently score many research ideas); (iii) input from other stakeholders (who agree on differential weights for the chosen priority-setting criteria according to a wider societal system of values) [16]–[18],[22]; and (iv) computation and discussion of the scores and analysis of the agreement between experts. The conceptual framework for the CHNRI methodology is shown in Figure 1. More detailed explanation has been published elsewhere [16]–[18],[22] and is also available in Table S1.

### (i) Input from Investors/Policy-Makers

The WHO CAH programme coordinated a large international exercise, involving more than 200 experts from about 80 different countries, to identify health research priorities that could directly tackle the main causes of global child mortality. The aim was to inform key global donors, public investors in health research, and international agencies on research investment policies that could support efforts to accelerate the progress towards MDG4. Thus, the context for this exercise was a short-term one, set within MDG4 and requiring an urgent and rapid progress in mortality reduction from childhood pneumonia. While defining this context, the WHO also recognised the importance of context-specific issues at local or regional levels, the large problem of pneumonia morbidity, and the beneficial effects of investments in the improvement of malnutrition and other cross-cutting and cross-sectoral issues [17],[18]. Further details are provided in Table S1.

### (ii) Input from Technical Experts

Individuals with a wide range of technical expertise and regional representation were recruited to participate. A large list of research questions was drafted by the technical expert group based on recent systematic reviews and a survey of experts. Initially, more than 500 questions were proposed. They were organised using the CHNRI framework for listing research questions, shown in Table S2. They were then compressed into a smaller number (158 questions) that still represented the broad spectrum of health research areas, topics, and instruments. The expert group then reviewed the questions, refining and reformulating them to allow the scoring. The final questions were sent to each technical group member for scoring. The criteria that were adopted were: (i) answerability (which captures the likelihood that each proposed research question can indeed be answered through a well designed study and in an ethical way, using the existing level of research capacity); (ii) likelihood of effectiveness; (iii) likelihood of deliverability, affordability, and sustainability; (iv) maximum potential impact on mortality reduction; and (v) predicted impact on equity. The CHNRI framework for scoring research questions is shown in Table S3 [17],[18]. Further details are provided in Table S1.

### (iii) Solicited Input from Other Societal Stakeholders

The five criteria for scoring may be perceived to be of varying importance and the value given to each criterion may vary with the perspective of stakeholders. For example, parents who have experienced a pneumonia-associated death may rate mortality reduction much higher than a research funder who may value answerability, or a health system planner who may be most concerned with deliverability. Hence, CHNRI undertook an exercise to poll a wide range of stakeholders and to weight the criteria based on values assigned by these stakeholders, as described elsewhere [22]. The weights applied in this exercise are explained in detail in Table S1.

### (iv) Computation of the “Research Priority Scores” and Average Expert Agreement

Completed worksheets were returned to the group coordinator. The overall research priority score (RPS) was computed as the mean of the scores for the five criteria [18], weighted according to the input from the stakeholders [22], according to the following formula:
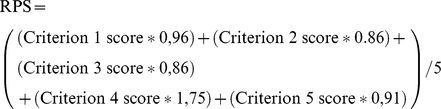



Average expert agreement (AEA) scores were also computed for each research question as the average proportion of scorers that gave the most common answer while scoring that particular research question. This is computed for each scored research investment option as:
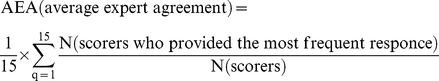
(where q is a question that experts are being asked to evaluate competing research investment options, ranging from 1 to 15). For further details regarding the choice of methods, agreement statistics, and interpretation, see Table S1.

## Results

Table 1 shows the top 10% of the 158 research questions, and Table S4 shows the complete list of ranks and scores. Both tables present the perceived likelihood that each research question will comply with each of the five chosen priority-setting criteria. Research questions from all four broad research domains (epidemiological research; health systems and policy research; research to improve the existing interventions; and research to develop new interventions) feature in the top 10% research questions. When the 30 questions with highest overall scores are considered (see Table S4), there is a predominance of research questions from the domain of “epidemiological research” (12/30) and health systems and policy research (8/30), while a smaller number came from the domain of “research to improve the existing interventions” (6/30) and “research to develop new interventions” (4/30). These results reflect the context of the exercise, i.e. expectation of short to medium term impact, within 5–10 years. This short time frame benefited epidemiological questions to assess and confirm the value of existing and available cost-effective interventions; health systems and policy research to identify key obstacles to delivery of those interventions on a larger scale; and optimising the use of those interventions (alone or in combination) in different contexts. The highest ranked questions address issues related to improving current case management and immunisation interventions, including systems-based approaches. The highest ranked issue is the study of barriers to care-seeking, an issue that is rarely given high funding priority by international agencies.

**Table 1:** The top 10% of research questions according to their achieved research priority score (RPS), with average expert agreement (AEA) related to each question.

Rank	Proposed Research Question	Res. Type	Answerable?	Effective?	Deliverable?	Burden Reduction?	Equitable?	AEA (%)	RPS (weighted)
1	Study the main barriers to health care seeking and health care access for children with pneumonia in different contexts and settings in developing countries	HPSR	89	93	88	57	92	73.3	79.7
2	Identify the key risk factors predisposing to the development of severe pneumonia and identify children who require hospitalisation	EPI	99	94	84	57	78	76.3	78.7
3	Study the main barriers to increasing coverage by available vaccines—Hib vaccine and pneumococcal vaccine—in different contexts and settings	HPSR	87	90	89	52	90	75.6	76.9
4	Study whether the coverage by antibiotic treatment can be greatly expanded in safe and effective ways if it was administered by community health workers	HPSR	82	86	90	52	97	75.2	76.8
5	Study the main barriers to increasing demand for/compliance with vaccination with available vaccines in different contexts and settings—for measles and pertussis vaccines, Hib vaccine, and pneumococcal vaccine	HPSR	90	86	85	51	94	73.3	76.5
6	Assess the effectiveness of new conjugate pneumococcal vaccines in reduction of childhood pneumonia morbidity and mortality in different settings	EPI	95	96	78	49	77	74.8	74.4
7	Identify the key bacterial and non-bacterial pathogens associated with childhood pneumonia morbidity and mortality at the global level in HIV- and non-HIV-infected children	EPI	88	83	78	51	83	71.1	72.7
8	Can community volunteers be trained to adequately assess, recognise danger signs, refer, and treat ARI?	RIEI	91	88	81	38	96	73.0	72.3
9	Study the capacity of health systems worldwide to correctly diagnose and manage childhood pneumonia, and obstacles to correct diagnosis and case management in developing country setting	HPSR	76	75	90	50	90	68.1	72.0
10	Identify cost reduction mechanisms for conjugate vaccines (e.g., regional purchasing consortia, private-public partnerships, and novel funding mechanisms)	RIEI	84	89	88	43	82	70.7	71.9
11	Identify systems capacity to provide (and main barriers to increase) availability of oxygen in health facilities	HPSR	92	94	75	41	85	72.2	71.6
12	Development of low-cost conjugate vaccines for pneumococcus	RDNI	84	85	83	51	75	65.2	71.6
13	Assess the role of micronutrient and macronutrient deficiencies in morbidity and mortality of childhood pneumonia.	EPI	94	88	73	43	85	73.0	71.3
14	Assess the effectiveness of high coverage, community-based antibiotic treatment for pneumonia on mortality in immunised and non-immunised populations	EPI	82	71	77	54	87	65.6	71.3
15	Identify funding mechanisms for continuous supply of conjugate vaccines (e.g., regional purchasing consortia, private-public partnerships, and novel funding mechanisms)	RIEI	83	80	91	46	80	64.4	71.2
16	Identify factors that affect implementing and sustaining WHO ARI management strategy	RIEI	84	87	96	36	83	67.8	70.9

ARI, acute respiratory infection; EPI, epidemiological research; HPSR, health policy and systems research; RIEI, research to improve existing interventions; RDNI, research to develop new interventions.

Research questions seeking to develop new interventions had only four representatives among the 30 highest ranked questions. This is not surprising given the short specified time frame (i.e., 5–10 years) by when it is really difficult to envisage new interventions that could have substantial impact. The four ideas that were strongly encouraged by the experts were development of: (i) low-cost conjugate vaccines for pneumococcus; (ii) low-cost cross-protective common protein vaccines for pneumococcus; (iii) combination vaccine against common bacterial pathogens of acute lower respiratory infections (ALRIs); and (iv) a new approach to culture-appropriate health education on health-seeking behaviour change. Among the bottom ranked 30 research options, the majority proposed development of entirely new interventions (20/30). In addition, six from the domain of “epidemiological research”, and four from the domain of “improvement of existing interventions” were given low priority. In the large majority of cases, the main reason for this was minimal, or entirely non-existent, optimism towards their possible impact on reduction of pneumonia within the context defined above (i.e., by 2015). This was coupled with concerns over effectiveness and deliverability of many of the proposed new interventions, such as anti-inflammatory agents, antioxidants, or other ideas, leading from fundamental molecular research of uncertain answerability and effectiveness that seeks to identify novel disease mechanisms and approaches to treatment. Another common concern was that they would be the least likely to improve equity, at least by the year 2015. For example, new interventions are very likely to be initially available only to those who can afford them.

Good discrimination between the levels of agreement among the scorers on the priority of the 158 questions was achieved by calculating AEA (Table 1; Table S4). The scores ranged from 46.7% to 76.3%, indicating the proportion of scorers that gave the most common answer to an average question they were asked in relation to a specific research investment option. AEA values are also presented for the top 10% of research questions in Table 1. Generally, the questions over which the greatest level of overall agreement was observed among the experts were those that also achieved very high overall research priority scores. The greatest points of controversy were the research questions related to development of entirely new interventions or some controversial topics (e.g. antiviral drugs, exposure to cold air, the role of air pollutants or combustion of biomass fuels, transdermal delivery of antibiotics, or genetically modified crops for improved nutrition).

The scores given to all 158 research questions from individual experts and their level of agreement for each research question are presented in Table S5. The full list of technical experts who were invited to participate, their expertise, and reasons for non-participation from those who declined are presented in Table S6. It is difficult to suimultaneously discuss strengths and weaknesses of many proposed research questions that were ranked in the middle of the list. Generally, these comprised a very broad mix of ideas of possible novel interventions and diagnostic tests of uncertain answerability and effectiveness; health policy and systems research ideas with a very limited potential impact on overall mortality reduction; support for new ideas that may not be affordable, sustainable, or improve equity; and improvements to existing interventions of uncertain deliverability and improved effect on mortality. Table S4 offers many examples of such research proposals.

The results of this exercise, which involved a substantial number of researchers active in studying the problem of childhood pneumonia, exposed how entirely different research questions can be considered research priorities depending on the criterion used. Box 2 shows the three highest scoring research questions within each of the five priority-setting criteria used. The research that would be most *answerable* is related to determining risk factors for severe pneumonia and referring sick children to a hospital. This question was also among those most likely to be effective, and carrying the greatest potential for disease burden reduction. Other highly answerable questions were improving the definition of an episode in a community and quantifying the problem of antibiotic resistance. The ideas that were considered most likely to be *effective* were studies to assess effectiveness of new conjugate pneumococcal vaccines in different contexts and studying health systems capacity to provide oxygen. The questions that would contribute to *improved outreach and delivery* were those studying factors that affect implementing and sustaining WHO's acute respiratory infections management strategy, studying the main barriers to increase coverage by available vaccines, and assessing the effectiveness of existing WHO treatment algorithms and guidelines. The *greatest potential for disease burden reduction* was assigned to research studying the main barriers to health care seeking and access, and the development of combo-vaccines against common bacterial pathogens. Research that would contribute mostly to *improving equity* was a study of expanded diagnosis, referral, and antibiotic treatment in a safe and effective way through community health workers' training, and evaluating culture-appropriate health education and public health messages on health-seeking behaviour change and hospitalisation.

**Box 2. The Three Highest Scoring Research Questions within Each of the Five Priority-Setting Criteria (CHNRI Scores Can Range from 0 to 100)**

**ANSWERABILITY**

1. Identify the key risk factors predisposing to the development of severe pneumonia and identify children who require hospitalisation (99/100)
2. Measure and compare the burden of pneumonia using existing WHO definition and newer alternate definitions of ALRI/clinical pneumonia that use X-ray or laboratory diagnostics and/or correct for diseases that mimic the presence of pneumonia (96/100)
3. Measure the frequency of antibiotic resistance among cases of pneumonia caused by common respiratory bacterial pathogens (96/100)

**EFFECTIVENESS**

1. Assess the effectiveness of new conjugate pneumococcal vaccines in reduction of childhood pneumonia morbidity and mortality in different settings (96/100)
2. Identify the key risk factors predisposing to the development of severe pneumonia and identify children who require hospitalisation (94/100)
3. Identify systems capacity to provide (and main barriers to increase) availability of oxygen in health facilities (94/100)

**DELIVERABILITY**

1. Identify factors that affect implementing and sustaining WHO's acute respiratory infections management strategy (96/100)
2. Study the main barriers to increase coverage by available vaccines—measles and pertussis vaccines—in different contexts and settings (94/100)
3. Assess the effectiveness of existing WHO treatment algorithms and guidelines on preventing pneumonia-related deaths, unnecessary referrals, and unnecessary antibiotic use (93/100)

**MAXIMUM POTENTIAL FOR MORTALITY REDUCTION**

1. Identify the key risk factors predisposing to the development of severe pneumonia and identify children who require hospitalisation (57/100)
2. Study the main barriers to health care seeking and health care assess for children with pneumonia in different contexts and settings in developing countries (57/100)
3. Development of combo-vaccine against common bacterial pathogens of ALRI (57/100)

**EQUITY**

1. Study whether the coverage by antibiotic treatment can be greatly expanded in a safe and effective way if it was administered by community health workers (97/100)
2. Can community volunteers be trained to adequately assess, recognise danger signs, refer, and treat acute respiratory infections? (96/100)
3. Investigate efficacy of the impact of culture-appropriate health education and public health messages on health-seeking behaviour change, hospitalisation, and mortality from childhood pneumonia (95/100)

Another sub-analysis that was allowed by the CHNRI process was evaluating the research ideas related to increased oxygen provision, which has often been a point of disagreement between donors, researchers, and implementors. Table 2 suggests that health systems research to improve availability of oxygen in health facilities and on the (cost) effectiveness of pulse oximeter technology should be given high investment priority within the short-term context; research on improving oxygen concentrator and other related technology be given medium priority; and research to define thresholds and improve user acceptability be given low priority. The exercise also illustrates the potential of this simple structured scoring system to give clear prioritisation among research options within a narrow research field and to give guidance on strengths and weaknesses of individual research questions to research policy-makers; in doing so, it limits individual biases by drawing together a larger number of experts from different backgrounds.

**Table 2:** An example of oxygen-related questions.

Rank	Proposed Research Question	Answerable?	Effective?	Deliverable?	Burden Reduction?	Equitable?	AEA (%)	RPS (weighted)
11	Identify systems capacity to provide (and main barriers to increase) availability of oxygen in health facilities	92	94	75	41	85	72.2	71.6
22	Determine effectiveness and cost-effectiveness of pulse oximeter on the use of oxygen to treat pneumonia and prevent deaths	89	81	75	35	77	67.4	66.0
58	Research to make oxygen concentrators further reduced in size and to improve their reliability and length of lifetime without maintenance	77	76	80	29	79	63.0	62.0
64	Research of improving oxygen concentrator technology to make it independent of electricity supply	89	70	75	27	80	65.2	61.9
70	Research to make technology related to oxygen interventions more robust and easily deliverable in both community settings and clinical practice	79	69	68	34	79	58.5	60.9
71	Optimise community-based oxygen therapy treatment of lower respiratory infections and assess its effectiveness	79	80	59	29	86	62.2	60.8
97	Define the criteria (threshold) at which treatment with oxygen improves survival	74	78	65	22	75	58.1	56.4
125	Identify strategies to improve acceptability of oxygen usage for children by guardians	79	55	66	9	67	53.3	48.1
144	Investigate if inhaled pulmonary vasodilatators synergise with oxygen and improve outcomes from very severe pneumonia	77	38	39	10	61	56.3	40.1

Several of the experts implied an apparent discrepancy in the perceived importance of oxygen delivery between the donors for health research, technical experts, and the implementers and programme leaders. The final ranks for the nine research questions related to oxygen research spread from 11th to 144th (among 158 suggested ideas). The ratio between highest and lowest scores varied widely across criteria: answerability (1.24), effectiveness (2.47), deliverability (2.05), impact of disease burden (4.56), impact on equity (1.39), and overall RPS (1.79). This example shows how a focus on addressing e.g., “oxygen research for pneumonia” would be too broad, and that prioritisation should be made between more specific research ideas to be meaningful.

## Discussion

The highest ranked questions in our priority-setting exercise address issues related to improving current case management and immunisation interventions, including systems-base approaches. This is not surprising, given that the context of the exercise was defined with a very short time frame (5–10 years), to which political commitment has been made through the support for the idea of MDGs. It is of interest that the highest ranked issue is the study of barriers to care-seeking, an issue that is rarely given high funding priority by international agencies. The process clearly showed how different research ideas can be seen as priorities based on different criteria, but also how some research questions satisfy most criteria and should represent apparent research priorities.

In this paper, we were primarily interested in research priorities that have a potential to reduce mortality from childhood pneumonia globally, thus contributing to achievement of MDG4. According to the most recent estimates, more than 99% of all pneumonia deaths occur in low- and middle-income countries. Because of this, addressing pneumonia deaths in wealthy countries by health research would not carry any potential to contribute to the main aim of our paper, and this is why the research on pneumonia in the high-income context hasn't been discussed. Furthermore, it takes a considerable amount of time to translate the outcomes of health research into interventions that, when rolled out, would indeed achieve measurable impact on the burden of any disease at the global level within a short time frame. This is why the proposed research agenda presented in Table 1 should merely be regarded as investments to accelerate progress toward the MDGs and beyond.

### Towards Transparent and Systematic Priority Setting In Global Health Research

The CHNRI methodology is a serious attempt to characterise many issues in the highly complex process of research investment priority setting; however, its validity is surely imperfect. For example, some good ideas (“research investment options”) may not have been included in the initial list of research options. Some ideas might be included due to excessive media interest. The conclusions represent the opinion of a limited group of involved people. Those and other possible biases and limitations of the method are described and discussed in greater detail in Table S1. Nevertheless, the method has rapidly become the most frequently applied tool to set research priorities at all levels, because it is very cheap and practical, simple to apply via e-mail, transparent and replicable, the output is intuitive and easily understood, and it has been validated and improved through many exercises over the past several years. We believe that it is important to use systematic and transparent methods and processes, and large expert groups, to keep exposing the strengths and weaknesses of different approaches in global health research. This should keep the focus of the donors on the areas where funding is most needed, for as long as the progress in reaching MDGs becomes truly satisfactory, and prevent it from drifting into other areas for which there is a lot of new advocacy, but not much evidence. We feel that the research community has a responsibility to expose strengths and weaknesses of the many competing ideas through transparent processes, and thus to reassure both the donors and the end users of health research investments that they should persist in supporting the activities with true potential to make a difference and save lives. Thus, the main goal of this paper was not to state the obvious, but rather to expose strengths and weaknesses of many competing existing and emerging research ideas. This should reassure the broad global health community on the choices that could be concluded reasonably quickly, and lead to interventions that would be likely to demonstrate measurable impact within a shorter time frame.

### A Need for Coordinated, Evidence-Based, and Equitable Research Investment Policies

The amount of funding available today for health research globally is unprecedented and the research investment market has been growing steadily over the past decade [23]. However, large inequities exist between amounts invested in different conditions that contribute to the global burden of disease. For example, while research on diabetes type 2 receives more than US$100 per DALY, research on pneumonia receives less than US$5 per DALY [14],[15],[23]. Perhaps a more pressing issue is the way in which the risk of investing in different health research domains is managed today. Long-term strategic investments in basic research, which are usually seen as highly uncertain, but also potentially highly profitable, may be justified in cases of chronic diseases, because those diseases can already be controlled by changes in diet and lifestyle and do not cause imminent threat to life. However, the situation with childhood diseases such as pneumonia and diarrhoea is quite different. Those two diseases combined continue to cause more child deaths each year worldwide than annual deaths attributable to smoking in all ages, or twice as many annual deaths as HIV/AIDS globally [13]. The persisting high mortality from pneumonia in the presence of existing cost-effective interventions and available resources to implement them represents a continuing scandal [13],[24],[25]. Given the consequences of the disease in terms of persisting child mortality, the level of urgency in dealing with this problem is very different than for other chronic diseases that contribute heavily to DALYs [13]. We believe that this should be reflected in global health research policies and investment strategies.

Investment in global health research today would benefit from consensus on the context, investment strategies, and coordination to achieve significant reduction of the disease burden in the foreseeable future—both among the investors, policy-makers, and researchers. The present exercise was designed to assist them all in making more informed choices on their investments in health research on pneumonia by exposing the risks and potential benefits associated with a broad spectrum of health research options. The expected “profit” from investments is associated with generating new knowledge that can be translated into development of new (or improvement of existing) interventions that are effective, deliverable, affordable, and can reduce the existing burden of disease and disability in an equitable way. The risk is associated with research that is not likely to satisfy some of those criteria. Investors' preference for high-risk investment in health research is particularly questionable when it is occurring in a context that requires urgent progress, such as childhood pneumonia [13]. The focus on complex challenges of implementation (i.e., improving health systems, training health workers, including poorly educated village health workers, improving drug supply and delivery at the community level, etc.), which the exercise highlighted, was reflected in many research questions being ranked near the top of the list of overall priorities.

The implementation of the CHNRI methodology showed that, within the context of MDG4, a better balance should be achieved between specific domains of health research. Along with continuing strategic long-term investments and new interventions, which represent “high risk - high-profit”, the CHNRI process suggested that more attention should also be given to health policy research, health systems research, operations research, and research that addresses political, economic, social, cultural, behavioural, and infrastructure issues surrounding the problem of child mortality. These domains of health research are rarely recognised as attractive by investors in health research because their results are unlikely to grab the headlines, get considered by journals with high impact factors, lead to patents or commercial products. Yet, they can generate new knowledge that can be exceptionally helpful in achieving real progress in mortality reduction.

This was an exercise aimed mainly at identifying research priorities to improve specific pneumonia prevention and management. If a broader policy context was more inclusive, policy research priorities to address underlying determinants (such as environment, nutrition, women's education, housing, social and political context, etc.) would surely also emerge as very important. A separate CHNRI exercise will investigate broader policies addressing underlying determinants of child health and cross-cutting issues that affect all major child diseases.

### Evaluation of the Process and Further Steps

With the emergence of the CHNRI methodology, several group leaders with the WHO spotted the opportunity to conduct an inclusive and systematic exercise to define child health research priorities globally that could help accelerate the progress towards MDG4. They conducted the process from WHO headquarters in Geneva, but included hundreds of external experts globally and collected their opinions. This paper is one of the five papers that resulted from this process, which has been seen as an example of a helpful, systematic, and transparent priority-setting exercise [19]–[24]. The members of the WHO CAH-based group were eventually happy to conclude that the identified priorities were in good agreement with the research that they already support at present. They emphasised the evaluation of existing interventions and the development and testing of new delivery approaches of existing interventions. They also highlighted the value of research on preventive measures, with research on new interventions being downplayed within the short-term context. But in reality, even these “shorter term” priorites (which can have more rapid impact on mortality reduction) would still take 10–20 years to fully explore in a developing country context, and past experiences have shown that each of these top priorities would likely entail a global research programme of a decade or more to see its impact fully realised. Following the completion of the exercise, a large donor conference called “Identifying priorities for Child Health Research to achieve MDG4” was held at the WHO in Geneva on March 26–27, 2009. More than 40 donor organisations were invited to choose and support some of the identified priorities. A publication that will summarise and discuss follow-up activities is in preparation.

## Conclusions

The context for this exercise was set within MDG4, requiring an urgent and rapid progress in mortality reduction from childhood pneumonia, rather than identifying long-term strategic solutions of the greatest potential. In a short-term context, the health policy and systems research to improve access and coverage by the existing interventions [25],[26] and epidemiological research to address the key gaps in knowledge [27] were highlighted as research priorities. These questions are mainly targeted at better understanding the barriers towards implementation, effectiveness, and optimisation of use of available interventions and programmes. If progress towards the reduction of global pneumonia mortality is to be improved by 2015, these are the research questions that are most likely to be of greatest importance. However, very few donors agencies recognise the importance of these domains of health research to readily invest in those options [14],[15]. The core group of CHNRI experts made several serious attempts to influence the key donors and point to this gap and serious imbalance in health research investing between long-term, strategic investments in basic research and support for instruments of health research that could contribute to mortality reduction in shorter term. This exercise, which involved much of the pneumonia research community, is the best example to date conducted at the global level.

## Supporting Information

Table S1 **The CHNRI methodology for setting priorities in health research investments.**
(DOC)

Table S2 **CHNRI's starting framework from which listing of many research options (level of 3–5-year research programme) and research questions (level of individual research papers) were being proposed by technical experts to systematically organise more than 500 research ideas and then develop a consolidated list of 158 research questions.**
(DOC)

Table S3 **Questions answered by technical experts to assign intermediate scores for each criterion to 158 competing research options (possible answers: Yes = 1; No = 0; Informed but undecided answer = 0.5; Not sufficiently informed = blank).**
(DOC)

Table S4 **The final list of priorities with intermediate and final priority scores for all 158 proposed research questions.**
(XLS)

Table S5 **The full list of scores given by 45 technical experts to each of the 158 research questions for each of the five priority-setting criteria.**
(XLS)

Table S6 **Composition of the group of technical experts.** An overview of expert selection, participation, and responses. All participation in this particular CHNRI exercise was voluntary and carried out without specific funding support. All the experts who were invited to participate in that exercise had a track record on research on childhood pneumonia, either as clinicians, epidemiologists, social scientists, or public health specialists.
(DOC)

## Abbreviations

AEA: average expert agreement
CHNRI: Child Health and Nutrition Research Initiative
DALY: disability-adjusted life year
GAPP: Global Action Plan for the Prevention and Control of Pneumonia
MDG: Millennium Development Goal
UN: United Nations
WHO: World Health Organization
