# Supplementary material for: Setting Research Priorities to Reduce Global Mortality from Childhood Pneumonia by 2015
Source: PLoS Med. 2011 Sep 27;8(9):e1001099. doi: 10.1371/journal.pmed.1001099 (PMC3181228; doi:10.1371/journal.pmed.1001099)
Supplement: Table S2 — CHNRI's starting framework from which listing of many research options (level of 3–5-year research programme) and research questions (level of individual research papers) were being proposed by technical experts to systematically organise more than 500 research ideas and then develop a consolidated list of 158 research questions. (DOC) [file pmed.1001099.s002.doc]

**Supplementary Table S2:** CHNRI’s starting framework from which listing of many research options (level of 3-5-year research program) and research questions (level of individual research papers) were being proposed by technical experts to systematically organize more than 500 research ideas and then develop a consolidated list of 158 research questions.

| RESEARCH INSTRUMENT | RESEARCH AVENUE | RESEARCH OPTION | RESEARCH QUESTION |
| --- | --- | --- | --- |
| Epidemiological research | Measuring the burden | Technical experts were invited to use categorization of research avenues and instruments to systematically propose a number of ‘research options’ within each of the avenues; ‘research options’ correspond to the level of 3-to-5-year research program | Technical experts were invited to propose a number of very specific ‘research questions’, corresponding to the title of individual research papers, within each of the ‘research avenues; eventually, after consolidation and removing of duplicate ideas, 158 such questions were retained for scoring |
|  | Understanding risk factors |  |  |
|  | Evaluating the existing interventions |  |  |
| Health policy and systems research | Studying capacity to reduce exposure to proven health risks |  |  |
|  | Studying capacity to deliver efficacious interventions |  |  |
| Research to improve existing interventions | Research to improve deliverability |  |  |
|  | Research to improve affordability |  |  |
|  | Research to improve sustainability |  |  |
| Research for development of new interventions | Basic research |  |  |
|  | Clinical research |  |  |
|  | Public health research |  |  |
