# Supplementary material for: Setting Research Priorities to Reduce Global Mortality from Childhood Pneumonia by 2015
Source: PLoS Med. 2011 Sep 27;8(9):e1001099. doi: 10.1371/journal.pmed.1001099 (PMC3181228; doi:10.1371/journal.pmed.1001099)
Supplement: Table S3 — Questions answered by technical experts to assign intermediate scores for each criterion to 158 competing research options (possible answers: Yes = 1; No = 0; Informed but undecided answer = 0.5; Not sufficiently informed = blank). (DOC) [file pmed.1001099.s003.doc]

**Supplementary Table S3:** Questions answered by technical experts to assign intermediate scores for each criterion to 158 competing research options (Possible answers: Yes=1; No=0; Informed but undecided answer: 0.5; Not sufficiently informed: blank).

*CRITERION 1:* Likelihood that research would lead to new knowledge (enabling a development / planning of an intervention) in an ethical way.

1. Would you say the research question is well framed and endpoints are well defined?

2. Based on: (i) the level of existing research capacity in proposed research; and (ii) the size of the gap from current level of knowledge to the proposed endpoints; would you say that a study can be designed to answer the research question and to reach the proposed endpoints of the research?

3. Do you think that a study needed to answer the proposed research question would obtain ethical approval without major concerns?

*CRITERION 2:* Assessment of likelihood that the intervention resulting from proposed research would be effective.

1. Based on the best existing evidence and knowledge, would the intervention which would be developed / improved through proposed research be efficacious?

2. Based on the best existing evidence and knowledge, would the intervention which would be developed / improved through proposed research be effective?

3. If the answer to either of the previous two questions is positive, would you say that the evidence upon which these opinions are based is of high quality?

*CRITERION 3:* Assessment of deliverability, affordability and sustainability of the intervention resulting from proposed research.

1. Taking into account the level of difficulty with intervention delivery from the perspective of the intervention itself (e.g. design, standardization, safety), the infrastructure required (e.g. human resources, health facilities, communication and transport infrastructure) and users of the intervention (e.g. need for change of attitudes or beliefs, supervision, existing demand), would you say that the endpoints of the research would be deliverable within the context of interest?

2. Taking into account the resources available to implement the intervention, would you say that the endpoints of the research would be affordable within the context of interest?

3. Taking into account government capacity and partnership requirements (e.g. adequacy of government regulation, monitoring and enforcement; governmental intersectoral coordination, partnership with civil society and external donor agencies; favourable political climate to achieve high coverage), would you say that the endpoints of the research would be sustainable within the context of interest?

*CRITERION 4:* Assessment of maximum potential of disease burden reduction.

*As this dimension is considered "independent" of the others, in order to score competing options fairly, their maximum potential to reduce disease burden should be assessed as potential impact fraction under an ideal scenario, i.e., when the exposure to targeted disease risk is decreased to 0% or coverage of proposed intervention is increased to 100% (regardless of how realistic that scenario is at the moment - that aspect will be captured by other dimensions of priority setting process, such as deliverability, affordability and sustainability)*

*Non-existing interventions**

*Maximum potential to reduce disease burden should be computed as "potential impact fraction” for each proposed research avenue, using the equation: PIF = [S_(i=1 to n)_ P_i_ (RR_i_-1)] / [S_(i=1 to n)_ P_i_ (RR_i_-1) + 1];*

*where PIF is “potential impact fraction” to reduce disease burden through reducing risk exposure in the population from the present level to 0% or increasing coverage by an existing or new intervention from the present level to 100%; RR is the relative risk given exposure level (less than 1.0 for interventions, greater than 1.0 for risks), P is the population level of distribution of exposure, and n is the maximum exposure level.*

*Existing interventions***

*Maximum potential to reduce disease burden should be assessed from the results of conducted intervention trials; if no such trials were undertaken, then it should be assessed as for non-existing interventions.*

*Then, the following questions should be answered:*

1. Taking into account the results of conducted intervention trials**, or for the new interventions the proportion of avertable burden under an ideal scenario*, would you say that the successful reaching of research endpoints would have a capacity to remove 5% of disease burden or more?

2. To remove 10% of disease burden or more?

3. To remove 15% of disease burden or more?

*CRITERION 5:* Assessment of the impact of proposed health research on equity.

1. Does the present distribution of the disease burden affect mainly the underprivileged in the population?

2. Would you say that either (i) mainly the underprivileged, or (ii) all segments of the society equally, would be the most likely to benefit from the results of the proposed research after its implementation?

3. Would you say that the proposed research has the overall potential to improve equity in disease burden distribution in the long term (e.g. 10 years)?
