# Supplementary material for: Setting Research Priorities to Reduce Global Mortality from Childhood Pneumonia by 2015
Source: PLoS Med. 2011 Sep 27;8(9):e1001099. doi: 10.1371/journal.pmed.1001099 (PMC3181228; doi:10.1371/journal.pmed.1001099)
Supplement: Table S6 — Composition of the group of technical experts. An overview of expert selection, participation, and responses. All participation in this particular CHNRI exercise was voluntary and carried out without specific funding support. All the experts who were invited to participate in that exercise had a track record on research on childhood pneumonia, either as clinicians, epidemiologists, social scientists, or public health specialists. (DOC) [file pmed.1001099.s006.doc]

**Supplementary Table S6.** Composition of the group of technical experts

An overview of expert selection, participation and responses. All participation in this particular CHNRI exercise was voluntary and carried out without specific funding support. All the experts who were invited to participate in that exercise had a track record on research on childhood pneumonia, either as clinicians, epidemiologists, social scientists or public health specialists.

| **N** | **Name** | **Expertise** | **Affiliation** |
| --- | --- | --- | --- |
| **Experts who contributed to the scoring process and are listed as co-authors of the paper** | | | |
| 1 | Igor Rudan | Epidemiologist, geneticist, public health specialist and researcher | Academic (The University of Edinburgh Medical School, UK); International organizations advisor |
| 2 | Shams El Arifeen | Clinician, public health specialist and researcher | Academic (ICDDR, B Centre for Health and Population Research, Dhaka, Bangladesh), International organizations advisor |
| 3 | Zulfiqar A. Bhutta | Clinician, public health specialist and researcher | Academic (The Aga Khan University, Karachi, Pakistan); Advisor to Ministry of Health, International organizations and International Paediatric Association |
| 4 | Robert E. Black | Clinician, epidemiologist, public health specialist and researcher | Academic (Johns Hopkins Bloomberg School of Public Health, USA); Advisor to International organizations |
| 5 | Abdullah Brooks | Clinician, public health specialist and researcher | Academic (ICDDR, B, Dhaka, Bangladesh; Johns Hopkins University, USA); Advisor to Ministry of Health and International organisations |
| 6 | Mickey Chopra | Clinician, public health specialist and researcher | Policy-maker at an International organization (Chief of Health, UNICEF, USA); |
| 7 | Trevor Duke | Paediatrician, public health specialist and researcher | Academic and Policy Maker (Centre for International Child Health , University of Melbourne; and Ministry of Health, Australia) |
| 8 | David Marsh | Public health specialist and researcher | Civil society (Save the Children, Amherst, USA); Advisor to Ministry of Health |
| 9 | Antonio Pio | Epidemiologist and public health specialist | Independent expert; Advisor to International Organizations |
| 10 | Eric Simoes | Paediatrician and researcher | Academic (The Children’s Hospital, Denver, USA); Advisor to Ministries of Health and International organizations |
| 11 | Giorgio Tamburlini | Clinician, public health specialist and researcher | Academic (Institute for Child Health IRCCS Burlo Garofolo, Trieste, Italy); Advisor to International organizations and International Paediatric Association |
| 12 | Evropi Theodoratou | Epidemiologist | Academic (The University of Edinburgh Medical School, UK); |
| 13 | Martin W. Weber | Paediatrician, public health specialist and researcher | Technical expert at International Organization (WHO Country Office, Jakarta, Indonesia) |
| 14 | Cynthia G. Whitney | Clinician, public health specialist and researcher | U.S. Government employee (Centers for Disease Control and Prevention, Atlanta, USA) |
| 15 | Harry Campbell | Paediatrician, epidemiologist, geneticist, public health specialist and researcher | Academic (The University of Edinburgh Medical School, UK); International organizations advisor |
| 16 | Shamim A. Qazi | Clinician, public health research and specialist, programme leader | Technical expert at International Organization (WHO Headquarters, Geneva, Switzerland) |
| **Experts who contributed to the scoring process and are listed under group co-authorship** | | | |
| 17 | Richard Adegbola | Clinician, public health specialist and researcher | Expert at the international donor organization (Bill and Melinda Gates Foundation) |
| 18 | Zrinka Biloglav | Epidemiologist | Academic (University of Zagreb Medical School) |
| 19 | Cynthia Boschi-Pinto | Epidemiologist | Technical expert at International Organization (WHO Headquarters, Geneva, Switzerland) |
| 20 | Lulu C. Bravo | Clinician | Academic (National Institutes of Health, University of the Philippines, Manila, Philippines) and Advisor to Ministry of Health and International organizations |
| 21 | Nigel Bruce | Clinician, epidemiologist, public health specialist and researcher | International Organization; Policy maker; Academic (Division of Public Health, University of Liverpool, Liverpool, United Kingdom) |
| 22 | Maria Regina Alves Cardoso | Epidemiologist and researcher | Academic (Faculty of Public Health, Sao Paulo University, Brazil) |
| 23 | A.L.A. da Cunha | Clinician and researcher | Academic (Federal University of Rio de Janeiro, Brazil) |
| 24 | Scott F. Dowell | Clinician, epidemiologist, public health specialist and researcher | U.S. Government employee (Centers for Disease Control and Prevention, Atlanta, USA); Advisor to Ministry of Health |
| 25 | Mike English | Clinician, public health specialist and researcher | Academic (KEMRI / Wellcome Trust Research Programme, Nairobi, Kenya); Advisor to Ministry of Health |
| 26 | Adegoke G. Falade | Clinician and researcher | Academic (College of Medicine, University of Ibadan, Nigeria) |
| 27 | Brian Greenwood | Clinician and researcher | Academic (London School of Hygiene and Tropical Medicine, London, United Kingdom) |
| 28 | Rana Hajjeh | Clinician, epidemiologist, public health specialist and researcher | U.S. Government employee (Centers for Disease Control and Prevention, Atlanta, USA); Advisor to Ministry of Health |
| 29 | Tabish Hazir | Clinician and researcher | Academic (Children’s Hospital, Pakistan Institute of Medical Sciences, Islamabad, Pakistan) and Advisor to Ministry of Health and International organizations |
| 30 | Patricia Hibberd | Clinican, epidemiologist, infectious diseases specialist and researcher | Academic (Division of Global Health, Department of Pediatrics, Massachussetts General Hospital) |
| 31 | Stephen Howie | Clinician and researcher | Academic (MRC Laboratories, Fajara, Banjul, The Gambia) |
| 32 | Prakash M. Jeena | Clinician and researcher | Academic (Nelson R. Mandela School of Medicine, University of Natal, Durban, South Africa) and Advisor to Ministry of Health |
| 33 | Karin Kallander | Epidemiologist, public health specialist and researcher | Civil society, Academic (Karolinska Institutet, Stockholm, Sweden) |
| 34 | Keith Klugman | Clinician, microbiologist, public health specialist and researcher | Academic (The Rollins School of Public Health, Emory University, Atlanta, USA); Advisor to Ministry of Health and International organizations |
| 35 | Shabir Madhi | Clinician and researcher | Academic (Respiratory and Meningeal Pathogens Research Unit, Chris Hani – Baragwanath Hospital, Bertsham Gauteng, South Africa); Advisor to International organizations |
| 36 | Kim Mulholland | Paediatrician, public health specialist and researcher | Academic (London School of Hygiene and Tropical Medicine, London, United Kingdom); Advisor to Governments and International oraganizations |
| 37 | Stephen K. Obaro | Public health specialist | Academic (Children’s Hospital of Pittsburgh, Pittsburgh, USA) |
| 38 | Stefan Peterson | Epidemiologist and public health specialist and researcher | Academic (Karolinska Institutet, Stockholm, Sweden); Advisor to Governments and International organizations |
| 39 | Zeba Rasmussen | Clinician and epidemiologist | Academic (Fogarty International Center, National Institutes of Health, Bethesda, USA) |
| 40 | Anna Roca | Epidemiologist and researcher | Academic (University of Barcelona, Spain) |
| 41 | H.P.S. Sachdev | Clinician, public health specialist and researcher | Private sector research organization (Sitaram Bhartia Institute of Science and Research, New Delhi, India); Academic; Advisor to Ministry of Health |
| 42 | Mathuram Santosham | Public health specialist and researcher | Academic (Johns Hopkins Bloomberg School of Public Health, Baltimore, USA); Advisor to Governments and International oraganizations |
| 43 | Anne Schuchat | Public health specialist and researcher | U.S. Government employee (Centers for Disease Control and Prevention, Atlanta, USA); Advisor to Ministry of Health and International organizations |
| 44 | Donald M. Thea | Clinician and researcher | Academic (Boston University School of Public Health, Boston, USA) |
| 45 | Paul Torzillo | Clinician and researcher | Academic (University of Sydney, Australia); Advisor to the Government |

DECLINED PARTICIPATION:

Of the other 26 experts invited to participate in this exercise, 22 did not respond, while four (2 epidemiologists, 1 microbiologist and one clinician) agreed to participate but later declined due to time constraint.
